# Case report: recurrent abdominal symptoms in a child with panhypopituitarism – there is always a differential

**DOI:** 10.1186/s13633-016-0037-3

**Published:** 2016-10-10

**Authors:** Laura Olbrich, Eva Schmidt, Ertan Mayatepek, Markus Vogel

**Affiliations:** 1 Department of General Pediatrics, Neonatology, and Pediatric Cardiology, University Children’s Hospital, Heinrich Heine University, Moorenstr. 5, 40225 Düsseldorf, Germany; 2 Department of Pediatric Oncology, Hematology and Clinical Immunology, University Children’s Hospital, Heinrich Heine University, Moorenstr. 5, 40225 Düsseldorf, Germany

**Keywords:** Panhypopituitarism, Familial Mediterranean fever, Recurrent abdominal pain, Adrenal insufficiency

## Abstract

**Background:**

We report the case of a 6 year old boy suffering from adenohypophysis aplasia as well as ectopic neurohypophysis and delayed diagnosis of familial Mediterranean fever (FMF).

**Case presentation:**

The boy was diagnosed with panhypopituitarism during the neonatal period and suffered from recurrent episodes during the following years suggesting infections. He also showed signs of adrenal insufficiency. Finally, at the age of 6 years, an additional diagnosis of familial Mediterranean fever (FMF) was clinically suspected and later confirmed by molecular analysis.

**Conclusion:**

The clinical pictures of panhypopituitarism and FMF can be overlapping. It is imperative to take a detailed and accurate history in order to find the right diagnosis, particularly a precise family history. In conditions like FMF an early diagnosis is crucial, as initiation of treatment with colchicine is important to prevent long-term complications due to amyloid fibril deposition.

## Background

Panhypopituitarism leads to growth hormone deficiency, secondary hypogonadism (LH/FSH deficiency), hypothyroidism (TSH deficiency) and adrenal insufficiency (ACTH deficiency) [1]. Symptoms of adrenal insufficiency are not specific and can mimic other processes. Patients with an acute crisis of adrenal insufficiency generally present with dehydration, hypotension, hypoglycemia, or altered mental status. Symptoms of persistent adrenal insufficiency include chronic fatigue, anorexia, nausea, vomiting, weight lcn [2].

Familial Mediterranean fever (FMF) is an autosomal recessive disorder that primarily affects Sephardic Jewish, Armenian, Turkish and Arab populations [3]. It is characterized by self-limited attacks of fever and peritonitis, pleuritis or synovitis recurring at irregular, unpredictable intervals and lasting from 24 to 48 h. Clinical manifestations during these attacks are mainly abdominal pain, chest pain and arthritis but also myalgia, skin manifestations and scrotal swelling [4, 5].

We report the case of a boy who was diagnosed with panhypopituitarism during the neonatal period and suffered from recurrent episodes during the following years suggesting infections while showing signs of adrenal insufficiency. Finally, at the age of 6 years, the additional diagnosis of familial Mediterranean fever (FMF) was clinically suspected and confirmed later by molecular analysis.

The clinical pictures of panhypopituitarism and FMF are overlapping, thereby leading to delay in diagnosis and suboptimal treatment if the underlying disorder is recognized incorrectly.

## Case presentation

**Fig. 1:**
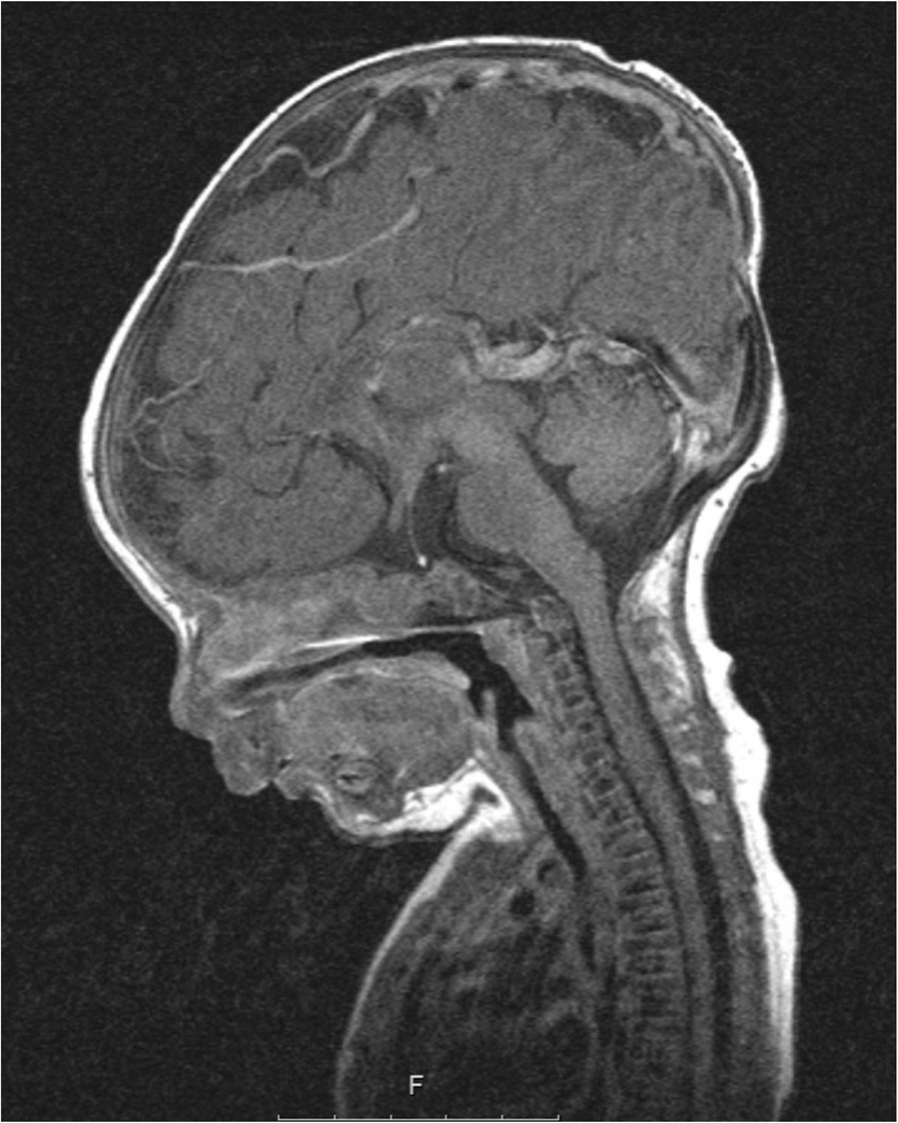
T1 sagittal MRI. Adenohypophysis is morphologically not definable, therefore, aplasia is suspected. The neurohypophysis presents as punctuate signal enhancement midsize of the hypophysis stem

A 6-year-old male of Turkish origin had been treated since birth because of adenohypophysis aplasia and ectopic neurohypophysis resulting in panhypopituitarism with secondary adrenal insufficiency, hypothyroidism and growth hormone deficiency. Due to persistent hypoglycemia, muscular hypotonia, hyperbilirubinemia and unclear respiratory and hemodynamic situations a cranial MRI (cMRI) had been performed, showing an aplasia of the adenohypophysis while the neurohypophysis presented as a punctiform enhancement in the pituitary stalk. Laboratory markers of the adrenal glands had been in concordance with the MRI findings showing a blood plasma cortisol level of <0.5 μg/dl and free cortisol <10 μg/24 h in urinalysis. ACTH level was <5 pg/ml supporting the diagnosis of hypopituitarism. Levels of Thyreoid-stimulating were <0.1 μIU/ml (0.35–4.5 μIU/ml), fT4 0.1 ng/dl (1.5–2.6 ng/dl), growth hormone (GH) <0.05 ng/ml (random sample, 0.43–2.40 ng/ml), Insulin-like growth factor binding protein 3 (IGFBP-3) 0.88 mg/l (0.80–3.60) and Insulin-like growth factor 1 (IGF-1) <25 ng/ml (41–313 ng/ml). Replacement treatment was initiated with hydrocortisone, L-thyroxine and growth hormone (Fig. 1).

However, beginning at the age of 2 years, recurrent episodes of abdominal pain, vomiting and raised temperature were noticed. As no infectious causes could be detected these episodes were attributed to the panhypopituitarism and were followed by changes in treatment. During a hospitalization for treatment optimization, the suspicion of a second underlying disease was raised. While being admitted the patient was suffering from relapsing episodes with abdominal pain, vomiting, headache and fever (38–40 °C). The parents later commented that the patient had been suffering from similar episodes for about 4 years. Such episodes occurred suddenly twice a month lasting for 1 day. While the attacks were interpreted as recurrent minor infections and symptoms of secondary adrenal insufficiency, hydrocortisone dosage was increased repeatedly. Despite the increased dosage no clinical improvement was observed. Clinical examination during those episodes showed a pale boy in pain with diffuse moderate abdominal pain, normal bowel sounds, and normal cardiopulmonary findings.

The following blood tests were normal during these episodes: complete blood count, liver and renal function tests and levels of ferritin and alpha-1-acid glycoprotein (AGP). Erythrocyte sedimentation rate (ESR) was 70 mm/h [normal value: <10 mm/h]. Furthermore infectious causes were excluded as all tests were normal, namely urine tests, stool microbiology and virology, and calprotectin in the stool.

There was no indication of chronic inflammatory bowel disease; however, the parents reported a female cousin of the patient who had been suffering from relapsing abdominal pain, joint pain and fever since the age of 6 years. Since the parents were consanguineous the clinical suspicion of familial Mediterranean fever was raised.

Laboratory diagnostics showed serum amyloid A (SAA) remarkably elevated at 1180 mg/l [normal value: <10 mg/l]. Molecular genetic testing revealed a homozygous M694V mutation of the MEFV gene (p.Met694Val, c.2080A>G), confirming the diagnosis of familial Mediterranean fever. Therapy with colchicine was started at a dose of 1 mg per day. Within 3 weeks SAA decreased to 4.20 mg/l and neither fever nor abdominal pain have occurred again within 6 months. During long-term follow-up the patient was not admitted again due to abdominal pain or episodes of recurrent fever; on long-term follow-up severity of symptoms was mild under colchicine treatment. Hormonal substitution was adjusted repeatedly for weight and clinical needs, and an interaction of both treatment regimens was not observed.

## Conclusions

As different endocrinological axes in panhypopituitarism are impaired, clinical symptoms vary and are not specific [1]. Adrenal insufficiency is the main cause of morbidity; the most dangerous complications are adrenal crises, which occur if cortisol levels do not meet the body’s acute demand [5], possibly leading to hypovolemic shock due to increased vascular permeability. Hence, acute adrenal insufficiency can present itself with symptoms such as acute dehydration, hypotension or hypoglycemia. The treatment goal, therefore, is achieving a homeostasis in the endocrinological axes, which is mainly substitutional. Adjustment of the glucocorticoid dosage is essential, especially during episodes of stress [6], such as infection, surgery, or major trauma.

Familial Mediterranean fever (FMF) is one the most common auto-inflammatory diseases characterized by recurrent episodes of polyserositis and fever, also known as familial paroxysomal polyserositis or recurrent hereditary polyserositis [7]. It is thought to be gene-associated as an autosomal recessive trait. Lately, doubts have been raised as cases of patients suffering from FMF with heterozygous status have been reported [8]. While the etiology is still uncertain, it is seen primarily in certain ethnic groups, such as Sephardic Jewish, Armenian, Turkish and Arab populations [5], although there have been reports of large cohorts in different populations, e.g. from Japan [9]. Affected pediatric patients present with heterogeneous symptoms. In a study by Majeed et al., more than 400 pediatric patients presented with abdominal pain in 81 % of exacerbations, chest pain (41 %), arthritis (42 %), severe myalgia (11 %), erysipelas-like skin manifestation (12 %), scrotal swelling (4 %), and recurrent episodic fever (3 %) [5]. Generally, during acute attacks serum markers of systemic inflammation are raised, and elevation of SAA is typical.

Diagnostic criteria include: (i) typical clinical manifestations, (ii) positive response to colchicine and (iii) genetic testing. FMF may subsequently be complicated by AA amyloidosis leading to chronic renal failure [10], which occurs in approximately 30 % of Sephardic Jews and 60 % of Turks with FMF [11]. The standard treatment for preventing attacks and amyloid deposition in patients with FMF is daily treatment with colchicine, reducing morbidity, particularly proteinuria, remarkably [12, 13]. Although the precise mechanism of colchicine in FMF treatment remains unknown, its beneficial role in treatment to induce remission has been substantiated in various studies. It should be introduced as soon as the diagnosis of FMF has been established and be continued for life. It has been reported to be beneficial even when amyloidosis is already present [12]. Alternative treatments for non-responders (approximately 5 %) are scarce; few studies have been conducted up to now. An anti-IL-1 treatment seems to be beneficial and studies showed the efficacy of Anakinra and Canakinumab in pediatric patients with FMF or juvenile idiopathic arthritis [14, 15].

FMF and symptoms secondary to panhypopituitarism are overlapping and characterized by rather non-specific symptoms (Table 1). In a study of 60 adult patients with adrenal insufficiency, the main causes of adrenal crises were gastrointestinal infection and fever [6]. Our patient presented repeatedly with abdominal pain, vomiting and headache. These symptoms were misinterpreted as recurrent minor infections for several years, resulting in an adrenal crisis.

**Table 1:** Symptoms of adrenal insufficiency and familial Mediterranean fever

Adrenal insufficiency	Familial Mediterranean Fever
Recurring abdominal pain	
Nausea and vomiting	
Fatigue	
Myalgia	
Anorexia and weight loss	
Dehydration	Recurring fever
Hypotension	Chest pain
Hypoglycemia	Joint pain
Altered mental status	
	Skin manifestation
	Scrotal swelling

One of the most helpful approaches in diagnosis is taking a detailed history. The Turkish origin of this patient, his parents’ consanguinity, and similar symptoms of the female cousin without panhypopituitarism finally led to the suspected diagnosis of FMF, which was confirmed by high levels of serum amyloid A and molecular genetic testing.

To our knowledge panhypopituitarism and FMF in the same pediatric patient have not been described before. Cases of hypoadrenal syndrome in patients with FMF have been described, mainly due to amyloid fibril deposition in adrenal glands [4]. Kadayifci et al. reported the coexistence of FMF and Addison’s disease in a young woman, supposing a similar immune mechanism of both diseases, suggesting a decreased suppressor T-cell-function, as seen in FMF and being a major disturbance in cellular immunity in Addison patients [4]. In our patient panhypopituitarism had been diagnosed already during the neonatal period. Due to persistent hypoglycemia, muscular hypotonia, hyperbilirubinemia and unclear respiratory and hemodynamic situations, a cMRI had been performed, showing aplasia of the adenohypophysis. Laboratory markers of the adrenal glands had been in concordance with the MRI findings. The diagnosis of FMF could be clinically and genetically confirmed; a single cause of both conditions is highly unlikely, as is an underlying immune mechanism.

Overlapping clinical signs and symptoms of infection, adrenal insufficiency, as well as systemic inflammation, can delay diagnosis of autoimmune disorders such as FMF. In any case a detailed anamnesis is crucial, especially an accurate family history, which should be one of the focal points of every investigation.

Attacks should be prevented, particularly in patients with adrenal insufficiency, as they can be stressors triggering adrenal crises leading to further complications. Early diagnosis is important to avoid unnecessary and invasive diagnostics such as laparotomy or appendectomy. In addition, early diagnosis may prevent long-term complications such as amyloidosis, and colchicine treatment should be initiated as soon as possible.

## Abbreviations

ACTH: Adrenocorticotropic hormone
cMRI: Cranial Magnetic Resonance Imaging
FMF: Familial Mediterranean Fever
FSH: Follicle-stimulating hormone
LH: Luteinizing hormone
SAA: Serum amyloid A
TSH: Thyreoid-stimulating hormone
